# Intralymphatic immunotherapy with one or two allergens renders similar clinical response in patients with allergic rhinitis due to birch and grass pollen

**DOI:** 10.1111/cea.14138

**Published:** 2022-04-01

**Authors:** Lars Ahlbeck, Emelie Ahlberg, Janne Björkander, Caroline Aldén, Georgia Papapavlou, Laura Palmberg, Ulla Nyström, Pavlos Retsas, Patrik Nordenfelt, Totte Togö, Pål Johansen, Bo Rolander, Karel Duchén, Maria C. Jenmalm

**Affiliations:** ^1^ Allergy Center University Hospital Linköping Sweden; ^2^ Division of Inflammation and Infection Department of Biomedical and Clinical Sciences Linköping University Linköping Sweden; ^3^ Futurum, Academy of Health and Care Jönköping Sweden; ^4^ Department of Medicine County Hospital Ryhov Jönköping Sweden; ^5^ Department of Dermatology University Hospital Zurich University of Zurich Zürich Switzerland; ^6^ Division of Children's and Women's Health Department of Biomedical and Clinical Sciences Linköping University Linköping Sweden

**Keywords:** allergy, intralymphatic immunotherapy, hypersensitivity, rhinoconjunctivitis immunotherapy, intralymphatic, allergy

## Abstract

**Introduction:**

There is a need for a fast, efficient and safe way to induce tolerance in patients with severe allergic rhinitis. Intralymphatic immune therapy has been shown to be effective.

**Methods:**

Patients with severe birch and timothy allergy were randomized and received three doses of 0.1 ml of birch and 5‐grass allergen extracts (10,000 SQ units/ml, ALK‐Abelló), or birch and placebo or 5‐grass and placebo by ultrasound‐guided injections into inguinal lymph nodes at monthly intervals. Rhinoconjunctivitis total symptom score, medication score and rhinoconjunctivitis quality of life questionnaire were evaluated before treatment and after each birch and grass pollen season during three subsequent years. Circulating proportions of T helper subsets and allergen‐induced cytokine and chemokine production were analysed by flow cytometry and Luminex.

**Results:**

The three groups reported fewer symptoms, lower use of medication and improved quality of life during the birch and grass pollen seasons each year after treatment at an almost similar rate independently of treatment with one or two allergens. Mild local pain was the most common adverse event. IgE levels to birch decreased, whereas birch‐induced IL‐10 secretion increased in all three groups. IgG4 levels to birch and timothy and skin prick test reactivity remained mainly unchanged. Conjunctival challenge tests with timothy extract showed a higher threshold for allergen. In all three groups, regulatory T cell frequencies were increased 3 years after treatment.

**Conclusions:**

Intralymphatic immunotherapy with one or two allergens in patients with grass and birch pollen allergy was safe, effective and may be associated with bystander immune modulatory responses.

**Clinical Trial Registration:** EudraCT (2013‐004726‐28).

## Graphical abstract

Immunotherapy with three monthly injections with extracts containing each 1000 SQ‐U of birch pollen and 1000 SQ‐U of grass pollen, or either and placebo, in bilateral inguinal lymph nodes was safe and effective. One or two allergens rendered similar clinical response in patients with allergic rhinitis due to birch and grass pollen. Symptoms, medication and quality of life were all improved in all treatment groups. An increase in the proportion of activated Treg cells may explain the results.

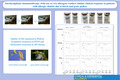

**Key Messages**

- Immunotherapy with three intralymphatic injections is safe and effective.
- One or two allergens render similar response in patients with allergic rhinitis due to birch and grass pollen.
- An increase in the proportion of activated Treg may render a bystander effect.

## 1 INTRODUCTION

Nearly 30% of the adult population of Sweden report allergic rhinitis.^1^ In addition, the prevalence of allergic sensitization, as determined as the presence of circulating IgE antibodies to birch, timothy, mugwort, cat, dog, horse, *Dermatophagoides pteronyssinus*, *Dermatophagoides farinae* and *Alternaria*, is up to 45% in Sweden and in many other European countries.^2^, ^3^ The total cost of allergic rhinitis in Sweden, with a population of 9.5 million (in 2014), has been estimated at €1.3 billion annually.^4^ Treating these patients with allergen immunotherapy (AIT) is cost‐effective.^5^ To date, AIT is the only treatment that affects the long‐term development of allergic rhinoconjunctivitis. It induces clinical tolerance primarily by stimulating regulatory T (Treg) cells, attenuating T helper 2 (Th2) responses and inducing blocking antibodies.^6^ Conventional AIT with subcutaneous injections is effective but consumes time and resources as it lasts more than 3 years and requires about 40 injections to complete.^7^, ^8^ Another method is sublingual immunotherapy (SLIT), where tablets containing allergens are given daily for 3 years^9^

Allergen immunotherapy for allergic rhinoconjunctivitis improves symptom, medication and combined symptom and medication scores (MS) in patients with allergic rhinoconjunctivitis.^10^ A randomized placebo‐controlled trial with subcutaneous immunotherapy (SCIT) with birch pollen allergen reduced the symptom score by 40% over placebo.^11^ In a review article of SCIT and SLIT trials, SCIT was reported to reduce nasal and ocular symptoms by 32–36% compared with placebo, whereas SLIT produced a reduction in 26–36% compared with placebo.^12^ Furthermore, both clinical and immunological bystander effects of specific AIT on other allergens have been reported in animal studies and one case report.^13^, ^14^, ^15^

In an open intralymphatic immunotherapy (ILIT) study,^16^ hayfever patients received three monthly allergen injections directly into the inguinal lymph nodes and an accumulated dose of 3000 standardized quantified units (SQ‐U) in contrast to approximately 3,000,000 SQ‐U with SCIT. Greater efficacy and safety and faster relief of symptoms were observed after ILIT than after SCIT. The study was followed by a few smaller studies of which five^17^, ^18^, ^19^, ^20^, ^21^ pointed in the same direction as the original trial, whereas one showed no benefit of ILIT.^22^ These studies were performed with timothy or birch pollen or both. Small ILIT studies with cat, house dust mite and dog have also shown positive clinical results.^23^, ^24^ Recently we performed a small open pilot study of 10 patients treated with ILIT for birch or grass pollen allergy. We concluded that ILIT was associated with improved quality of life, reduced symptoms and beneficial immunological changes.^21^ However, further studies are required to determine whether ILIT can induce clinical effects similar to those of SCIT concerning rhinitis symptoms and how ILIT affects immune responses.^25^

## 2 METHODS

### 2.1 Aim

The objective was to evaluate safety and efficacy after ILIT with one or two allergens: birch‐ or grass pollen or both. We also aimed to determine its immune modulatory effects including changes in spontaneous and allergen‐induced cytokine and chemokine production, and proportions of circulating T helper cell subsets.

### 2.2 Study design

A 3‐year double‐blind randomized clinical trial in 72 patients with rhinoconjunctivitis due to sensitization with birch and grass pollen allergens. The patients were given active treatment with birch or grass in one inguinal lymph node and active treatment with the other allergen or placebo in an inguinal lymph node on the other side. The study was approved by Regional Ethics Committee in Linköping (EPN number 2013/487‐31).

### 2.3 Study population eligibility criteria

**FIGURE 1:**
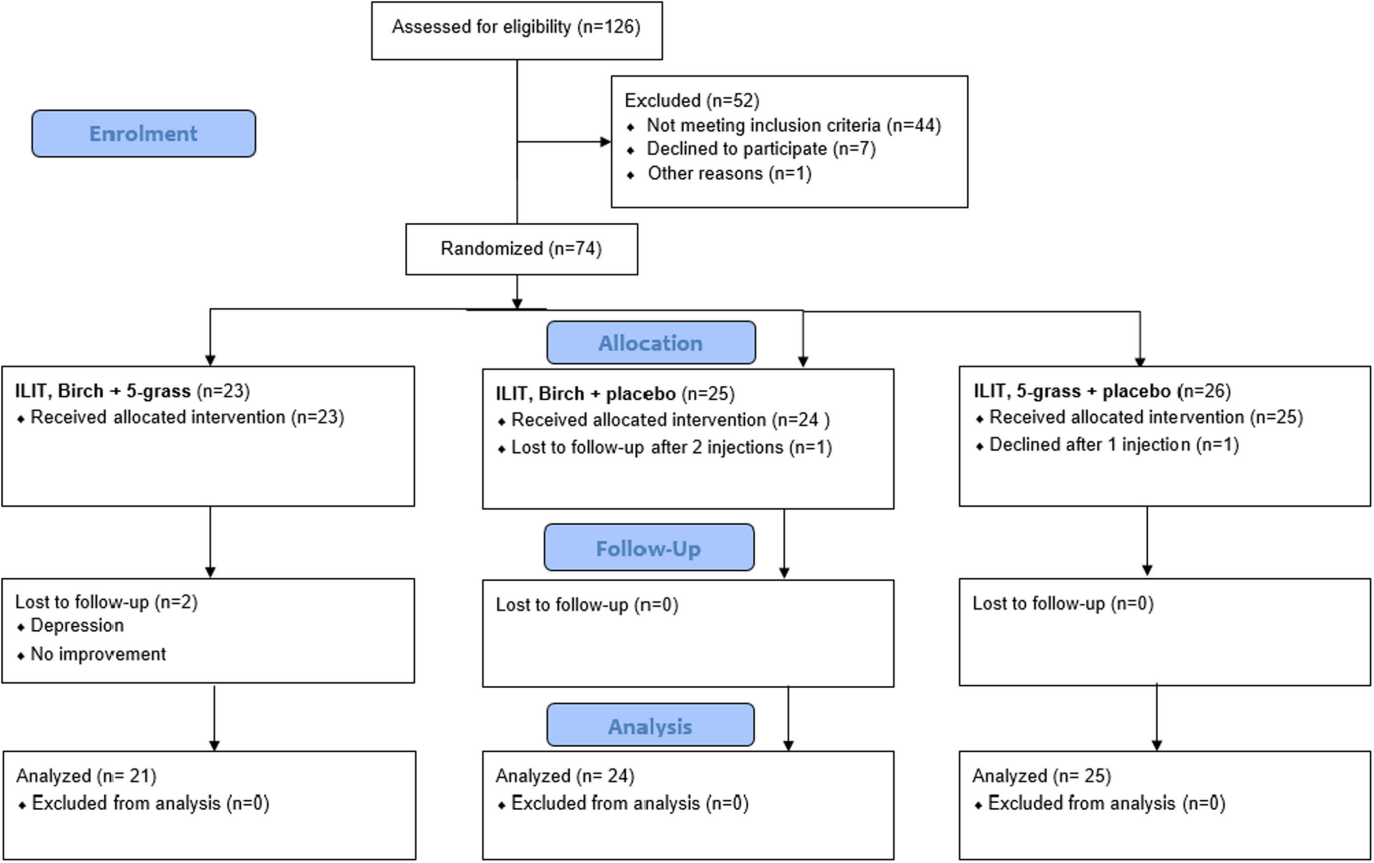
126 patients were assessed for eligibility. 52 were excluded. 74 patients were randomized to ILIT with three doses of birch‐ and 5 grass‐pollen allergen extracts, or either and placebo at monthly intervals. 72 patients received all injections. 70 patients remained for analyses 3 years after treatment

In all, 126 patients were assessed for eligibility. All patients signed written informed consent to participate in the study. Forty‐four did not meet the inclusion criteria, 7 withdrew consent before treatment and 1 was excluded for unknown reasons (Figure 1). Fifty‐seven patients with allergic rhinoconjunctivitis due to birch and timothy pollen allergens were randomized in 2014 and 17 patients 2015. The 74 patients, including 35 females, were 19–53 years old and had seasonal allergic symptoms to birch and grass (Table 1), whereof 28 were randomized and followed in the Department of Medicine, County Hospital Ryhov, Jönköping. All participants were given ILIT at Allergy Center, University Hospital, Linköping, Sweden. Their skin prick test was >3 mm and displayed IgE to birch and timothy >0.35 kU/L. Exclusion criteria were pulmonary disease other than asthma, asthma with <80% of predicted forced expiratory volume at the end of the first second (FEV1), use of more than 800 µg inhaled budesonide (or equivalent) per day, pregnancy, severe arterial hypertension, autoimmunity, cardiovascular, hepatic, renal, upper airway or metabolic disease, mental incapacity, alcohol abuse, medication interfering with immune response or beta‐blockers. From earlier studies we expected 8 out of 9 patients would improve at least 40%. With 40 active treated and 20 in the placebo group, with an alpha of 0.05 the power was calculated to 92%.

**TABLE 1:** Baseline characteristics by treatment group, presented as mean and standard deviation

	Birch + 5‐grass	Birch + placebo	5‐grass + placebo
*n*	23	25	26
Site (Linköping/Jönköping)	14/9	15/11	16/10
Female	11 (49%)	12 (48%)	12 (46%)
Mean age at study start	38.1 ± 10.2	36.8 ± 10.0	34.8 ± 9.9
Min/max age at study start	19.3/53.3	21.0/53.4	19.5/51.4
Other sensitizations^a^	2.6 ± 1.1	1.8 ± 1.3	2.7 ± 0.9
FEV1%	90.5 ± 10.0	97.3 ± 9.7	91.8 ± 7.6
FENO ppb	24.5 ± 27.0	22.2 ± 17.8	22.6 ± 15.7

Note

FEV1%: forced expiratory volume at the end of the first second, percent of predicted value.^35^, ^36^ FENO‐ppb: fraction of exhaled nitric oxide in parts per billion.

^a^ Number of other positive SPT with mugwort, cat, dog, horse, *Dermatophagoides pteronyssinus*, *Dermatophagoides farinae*, *Cladosporium*, *Alternaria* and *Aspergillus* extracts (Soluprick SQ Birch and Timothy, ALK‐Abelló, Hørsholm, Denmark).

### 2.4 Intralymphatic immunotherapy

The patients were randomized into three groups receiving three doses at 4‐week intervals of 0.1 ml of birch pollen allergen on aluminium hydroxide (10,000 SQ‐U/ml; ALK‐Abelló) and/or 0.1 ml of 5‐grass pollen allergen on aluminium hydroxide (10,000 SQ‐U/ml; ALK‐Abelló. 5‐grass is a mix of equal amounts of SQ‐U of *Alopecurus pratensis* (meadow foxtale), *Dactylis glomerata* (cocks’s foot), *Festuca pratensis* (meadow fescue), *Lolium perenne* (English ryegrass) and *Phleum pratense* (timothy). Each allergen dose was 1000 SQ‐U. A diluent from ALK was used as placebo. Thus the participants received two injections, one in each groin on three occasions. Patients were randomized in blocks of six, facilitated by Forum Östergötland. An unblinded nurse prepared and marked each syringe with a label providing randomization number, injection number and injection site. ILIT was administered by blinded clinicians at Allergy Center. Ultrasound‐led technique was used whereby a lymph node was punctured with a 27G (0.4 × 40 mm) needle. Histamine‐1 blocker desloratadine tablet 5 mg was given 15 min prior to the injections.

### 2.5 Primary outcome measures

Symptoms and drug consumption were primary outcome measures. Symptoms were validated based on the rhinoconjunctivitis total symptom score (RTSS) questionnaire.^26^ Drug consumption was measured using an MS questionnaire (see File S1). Medication was not provided by our study, but participants bought it themselves over the counter or prescribed as in normal health care. The RTSS and MS were recorded by the patients at the end of the birch pollen season (approximately June 1st) and at the end of the grass pollen season (approximately September 1st) before treatment and for the following 3 and 4 year’s altogether, no daily symptom score was recorded. The birch‐ and grass peak pollen seasons are separate in Sweden.

### 2.6 Safety assessment

Safety was assessed as the recording of adverse events from the time of the first injection to 3 years after the last injection. A research nurse called the patients to assess adverse events during the first 5 days after each injection. Safety laboratory parameters were assessed at screening, after the third ILIT injections and after the first pollen season following treatment (Table 2).

**TABLE 2:** Scheme of schedule procedures

	Screening	Randomization	Intervention			Follow‐up		
	Visit 1	Visit 2	Visit 3	Visit 4	Visit 5	Visit 6	Visit 7	Visit 8
Informed consent	x							
Phys. examination	x	x				x	x	x
Blood pressure, pulse, PEF	x	x	x	x	x	x	x	x
Safety tests^a^	x	x				x		
Immunol. tests^b^	x	x				x		x
SPT	x	x				x	x	x
Lung function	x	x				x	x	x
U‐HCG		x	x	x	x			
RQLQ, RTSS, MS^c^	x	x				x	x	x
CAPT^d^		x				x		x
AE		x	x	x	x	x	x	x
Concom. med	x	x	x	x	x	x		
Diary teaching ^e^	x				x	x	x	
ILIT			x	x	x			
Tel contact^f^			x	x	x			

Note

Visit 1: Pre‐ILIT, pre‐season. Visit 2: pre‐ILIT, post‐season. Visit 3; 7‐35d post visit 2. Visit 4: 28‐42d post visit 3. Visit 5: 28‐42d post visit 4. Visit 6: Fall year 1. Visit 7: Fall year 2. Visit 8: Fall year 3. All inclusion and exclusion criteria were checked at visits 1–5.

Abbreviations: ILIT, Intralymphatic immunotherapy; PEF, peak expiratory flow; SPT, skin prick test, U‐HCG, urine human chorionic gonadotropin (only in females).

^a^ Haematology: leukocytes, leukocyte differentiation (neutrophil, eosinophils, basophils and lymphocytes) haemoglobin and platelets at visits 1, 2 and 6. Coagulation blood tests at visits 1 and 2 (PK‐INR and APTT).

^b^ Total IgG and subsets (lgG1‐G4), total IgE, allergen‐specific IgE and IgG4, and other immunological tests.

^c^ RTSS, RQLQ and MS were measured after the birch pollen season (approx. Jun 1st) and after the grass pollen season (approx. Aug 1st)

^d^ The conjunctival challenge tests (CAPT) with timothy were performed according to the EAACI guidelines.^23^ Due to lack of extract from the company planned CAPT were not performed after the third pollen season.

^e^ The diary had space for description of the adverse events (AEs) since the last visit.

^f^ Two to five days after visit 3–5, telephone contact was made considering symptoms after the allergen injections.

### 2.7 Secondary outcome measures

Effects on quality of life were measured using the rhinoconjunctivitis quality of life questionnaire (RQLQ)^27^ was recorded by the patients at the end of the birch pollen seasons (approximately June 1st) and at the end of the grass pollen seasons (approximately September 1st) thus mirroring the last season rather than the last week. Skin prick test reactions (Soluprick SQ Birch and Timothy, ALK‐Abelló), allergen‐specific IgE and allergen‐specific IgG4 levels were analysed (ImmunoCAP ThermoFisher) before ILIT and in the fall the following 3 years. Conjunctival allergen provocation tests (CAPT)^28^ were performed with timothy (Aquagen SQ Timothy, ALK‐Abelló) before treatment and after the first pollen season after treatment. Due to lack of extract from the company planned CAPT were not performed after the third pollen season (Table 2).

### 2.8 Immune laboratory methods

Flow cytometry was used to analyse the CD4+ Th cell population in whole blood from the patients at randomization, and 1 and 3 years after completed ILIT. Peripheral blood mononuclear cells obtained from the patients at randomization and 1 year after ILIT were stimulated *in vitro* with birch and timothy allergen. Levels of IL‐5, IL‐10, IL‐13, IFN‐γ, CCL17 and CXCL10 were quantified using Luminex. For logistic reasons immune tests were only analysed from the 45 participants from the Allergy Center, University Hospital, Linköping, Sweden. For detailed methods, experimental protocols and statistical analyses, see Files S2 and S3.

### 2.9 Statistics

Descriptive statistics for RQLQ, RTSS and MS are presented in medians and percentiles (p25 and p75), and in the graphs with medians and 95% confidence interval. Paired comparisons over time were calculated with Friedman’s test and adjusted with the Bonferroni correction for multiple comparisons. Descriptive statistics for IgE, IgG4, SPT and CAPT are presented in mean values and standard deviation (SD). Paired comparisons over time were calculated with repeated measures ANOVA with Bonferroni confidence interval adjustment. The answers to the 28 questions in RQLQ were explored with an item analysis, rendering Cronbach's Alpha at 0.933; thus, the changes within the different domains of RQLQ were consistent. All analyses above were performed in SPSS version 25 (IBM Corp.).

All flow cytometry, cytokine and chemokine data were analysed using GraphPad Prism, version 8.3.1 (GraphPad software, Inc.) and non‐parametric tests were used. Comparisons at the different time‐points within the treatment groups were calculated using paired Wilcoxon signed ranks test. Unpaired Mann–Whitney *U* test was used to compare differences between the treatment groups at the different time‐points. The significance level was set at *p* < .05.

## 3 RESULTS

Seventy‐four patients were randomized to ILIT with three doses of 0.1 ml of either birch and placebo, 5‐grass and placebo or both birch and grass allergen extracts at monthly intervals. Seventy‐two patients received all injections (Figure 1). One patient was lost to follow‐up 2 years after treatment and his RQLQ and RTSS had been halved and the patient had not required or used anti‐allergic medication. Another patient was lost to follow‐up 3 years after ILIT, but 2 years after treatment the patient showed no improvement in RTSS, MS or RQLQ. Hence, 70 patients remained for analyses 3 years after treatment.

### 3.1 Symptoms and medication

**FIGURE 2:**
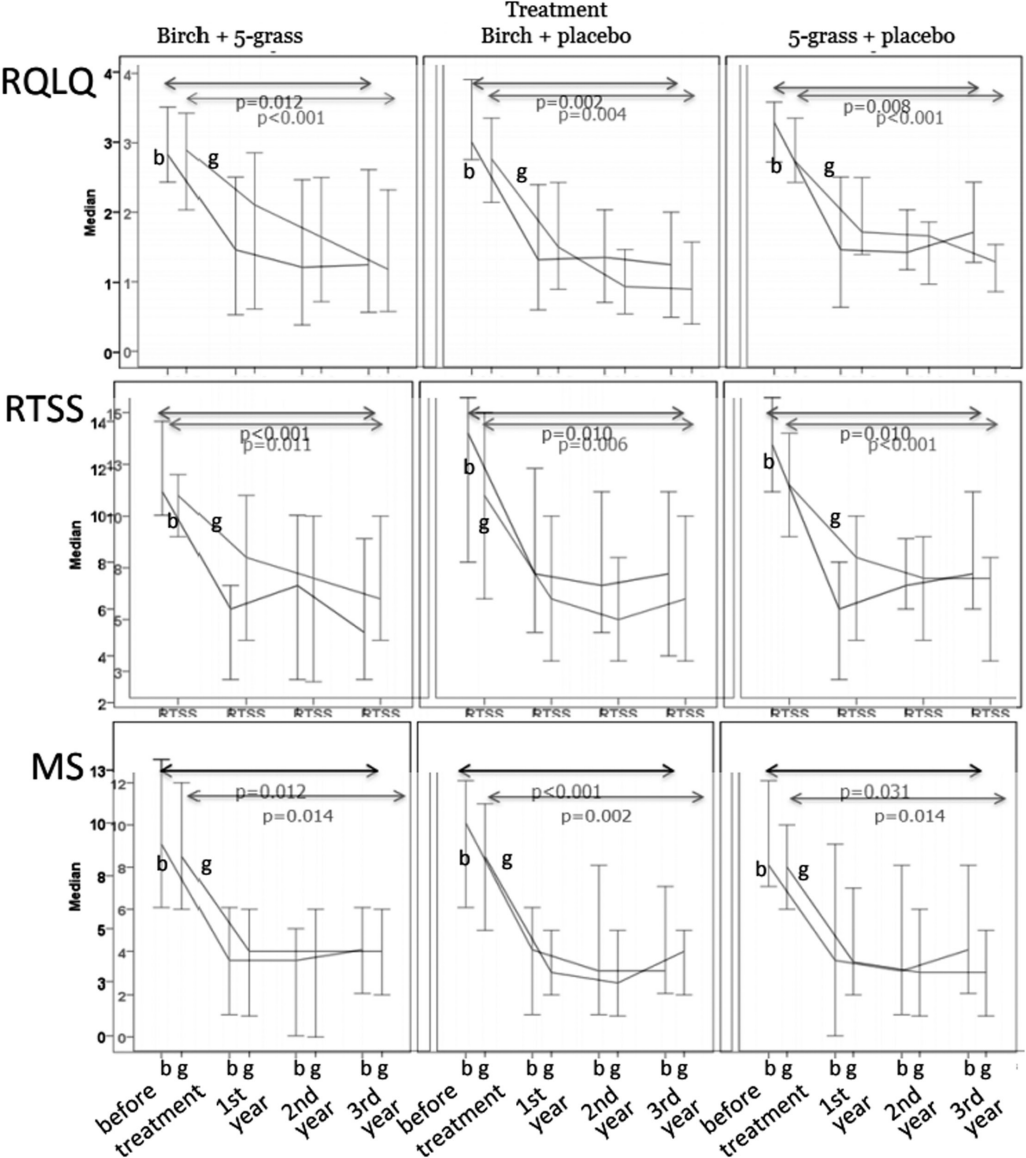
Quality of life measured as RQLQ, Symptoms measured as RTSS and medication measured as MS (see under Method section in the File S2). Results during birch pollen seasons (b) and grass pollen seasons (g) MS, medication score; RQLQ, Rhinoconjunctivitis Quality of Life Questionnaire; RTSS, Rhinoconjunctivitis Total Symptom Score

The symptoms measured by the RTSS and MS were significantly reduced 3 years post ILIT regardless of active allergen during the birch and grass pollen seasons. The reduction was already evident during the first season after ILIT, and the effect was sustained throughout the following 3 years. When combining all three groups, all having received one or two active allergens, RTSS was reduced from 12.2 to 7.4 (−39%) and from 11.2 to 6.5 (−42%) 3 years after treatment during the birch and grass pollen seasons, respectively (*p* < .05, Figure 2, and File S4). When combining MS data from all three treatment groups together, it was significantly reduced from 9.4 before ILIT to 4.9 (−48%) and from 8.6 to 4.4 (−49%) 3 years after treatment during the birch and grass pollen seasons, respectively (*p* < .01, Figure 2). For levels of RTSS and MS, see File S4). There were no differences in reduction in RTSS nor MS between the three groups. There were no sex‐related differences in improvement (data not shown). The patients receiving ILIT in 2014 and 2015 responded in a similar way (data not shown).

### 3.2 Adverse events

A total of 438 injections were given, of which 285 had an active substance containing allergen extract. Mild local pain at the injection site was the most common adverse event (AE) (Table 3). However, on three occasions, patients recorded severe pain from ILIT. One patient had moderate breathing problems without any fall in peak flow 30 min after the second ILIT (birch and 5‐grass) and received the third treatment without any AEs. One patient experienced breathing problem 2 h after physical activity, 4 days after the first injections and was relieved with salbutamol inhalations, antihistamine and oral corticosteroids. The remaining injections followed without breathing problems. During the follow‐up, 3 years after ILIT, nine patients reported severe AEs, diverticulitis, miscarriage, burn injury, disc hernia, abdominal pain, hysterectomy, concussion, chest pain and heart failure, but none was judged to be related to ILIT. One patient showed hypothyroidism with elevated levels of antithyroid peroxidase, and thyroid‐stimulating hormone receptor antibodies diagnosed in 2018, possibly related to the therapy given in 2014. No anaphylactic reaction was observed or reported.

**TABLE 3:** Cumulative data for adverse events

Organ system	Frequency	Adverse events
General symptoms and/or symptoms from the injection site	Very common (>1/10; 162 of the 216 visits, whereof 94 only on one injection site)	Immediate or late local reactions, swelling, pain at the injection
Immune system	Very common (>1/10: 29 of 216 visits)	Immediate or late systemic reactions, nasal congestion, itch, ocular itch, eczema
Immune system	Rare (1/1000–1/10,000; 0 of the 216 visits)	Anaphylaxis, anaphylactic chock

Note

Including 216 visits, hence 432 injections whereof 1/3 received ILIT with two allergens and 2/3 received ILIT with one allergen and placebo.

### 3.3 Health‐related quality of life

The impact on health‐related quality of life as measured by RQLQ was significantly reduced during the birch and grass pollen seasons, regardless of active ILIT with birch, 5‐grass or both allergens. When combining all three groups, the RQLQ score was significantly reduced from 3.15 to 1.50 (−52%) and from 2.82 to 1.25 (−56%) 3 years after treatment during the birch and grass pollen seasons, respectively (*p* < .01, Figure 2, and File S4).

### 3.4 Conjunctival allergen provocation tests, IgE, IgG4 and skin prick tests

Conjunctival allergen provocation test^28^ with timothy were performed before ILIT and the first year post ILIT, and showed a higher tolerance for increased concentrations of timothy pollen, in patients receiving ILIT with birch and 5‐grass (*p* < .05) and 5‐grass and placebo (*p* < .05), but not significant in patients who received ILIT with birch and placebo (*p* = .19, see File S4). The IgE levels to birch decreased from baseline to 3 years post ILIT from 25.45 to 17.71 kU/L (*p* < .01) in the group treated with birch and placebo; in the group treated with 5‐grass and placebo the IgE levels to birch decreased from 33.18 to 24.42 (*p* < .01). The levels of IgE to timothy decreased after ILIT with birch and placebo from 16.60 to 11.00 kU/L (*p* < .05); similar, but non‐significant differences were determined after ILIT with 5‐grass and placebo. IgE levels to both birch and timothy decreased slightly (*p* < .01 and <.05) after ILIT with both birch and 5‐grass. There was no significant decrease in the levels of total IgE (see File S4). Levels of IgG4 antibodies to birch and timothy remained unchanged in all three treatment groups, except for IgG4 levels to timothy, which increased from in mean 0.36 to 0.44 mg/L (*p* < .05) after ILIT with birch and 5‐grass (see File S4). Skin prick test for reactivity to birch and timothy allergens remained unchanged during the study period (see File S4).

### 3.5 Circulating T helper cell subsets

**FIGURE 3:**
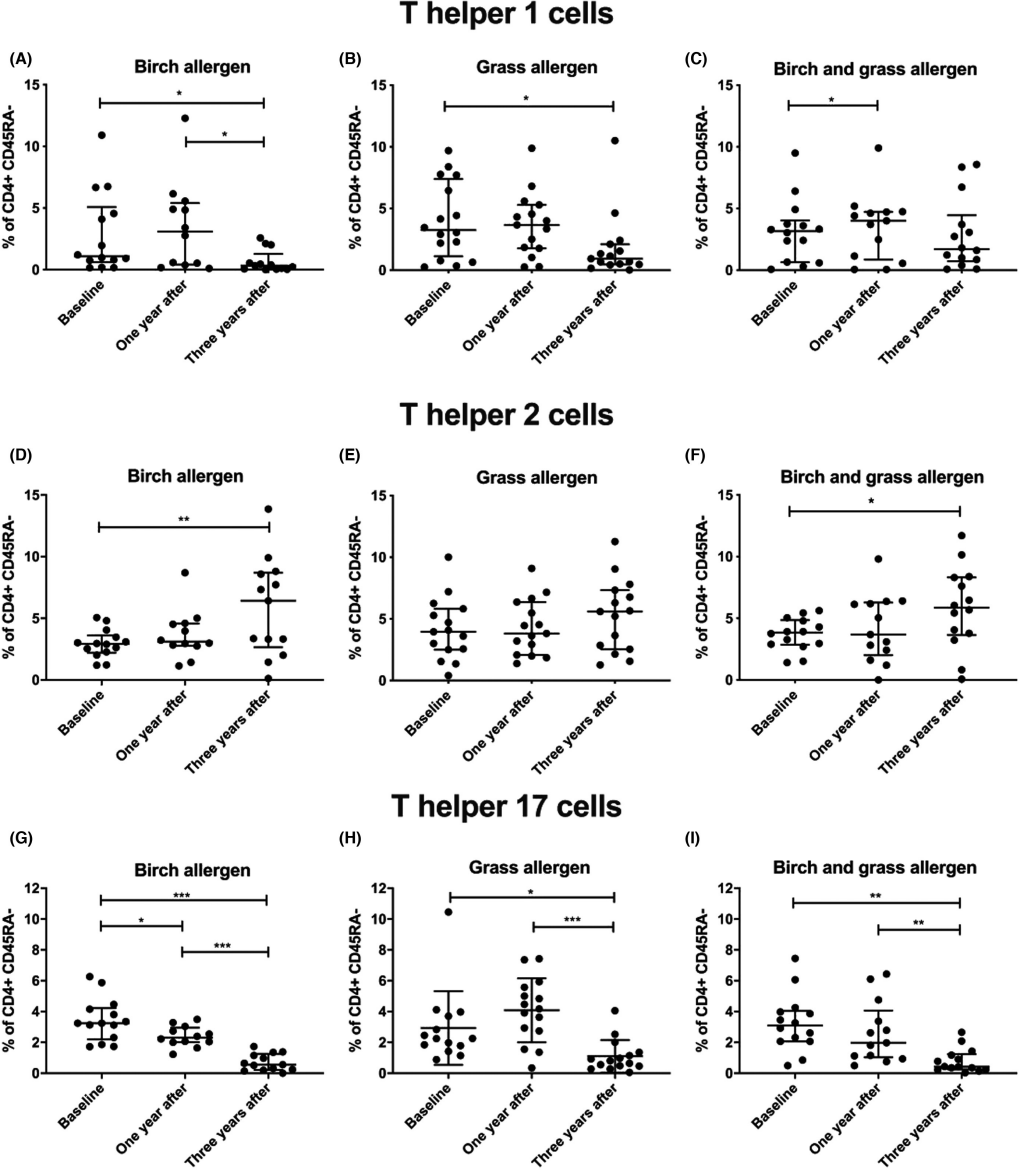
Proportion (%) of the T helper (Th) cells in the CD4^+^CD45RA^−^ memory populations after intralymphatic immunotherapy with birch and/or 5‐grass allergen. Blood samples were collected at three time‐points: screening, 1 year after and 3 years after treatment had finished. The proportion of Th1 cells, defined as CD3^+^CD4^+^CD45RA^−^Tbet^+^ cells, decreased over time after treatment in the groups receiving birch and placebo (A) and in the group receiving grass and placebo (B). A slight increase was observed between baseline and 1 year after treatment in the group receiving both birch and grass allergen (C). The proportion of Th2 cells, defined as CD3^+^CD4^+^ CD45RA^−^GATA3^+^ cells, increased between baseline and 3 years after treatment in the group receiving birch (D), not in the group receiving grass and placebo. Furthermore, Th2 cells increased 3 years after treatment in the group receiving both treatments (F). The proportion of Th17 cells, defined as CD3^+^CD4^+^ CD45RA^−^RORC^+^ cells, decreased between all time‐points in the treatment group receiving birch and placebo (G). The treatment groups receiving grass and placebo treatment (H) and birch and grass treatment (I) had similar changes; the proportion decreased between baseline and 3 years after treatment. A decrease was also observed between 1 year after and 3 years after treatment had finished. *p < .05; **p < .01; ***p < .001 from Wilcoxon signed rank tests. The lines indicate median and interquartile range (IQR, 25th and 75th percentile values). Only patients randomized in Linköping included

**FIGURE 4:**
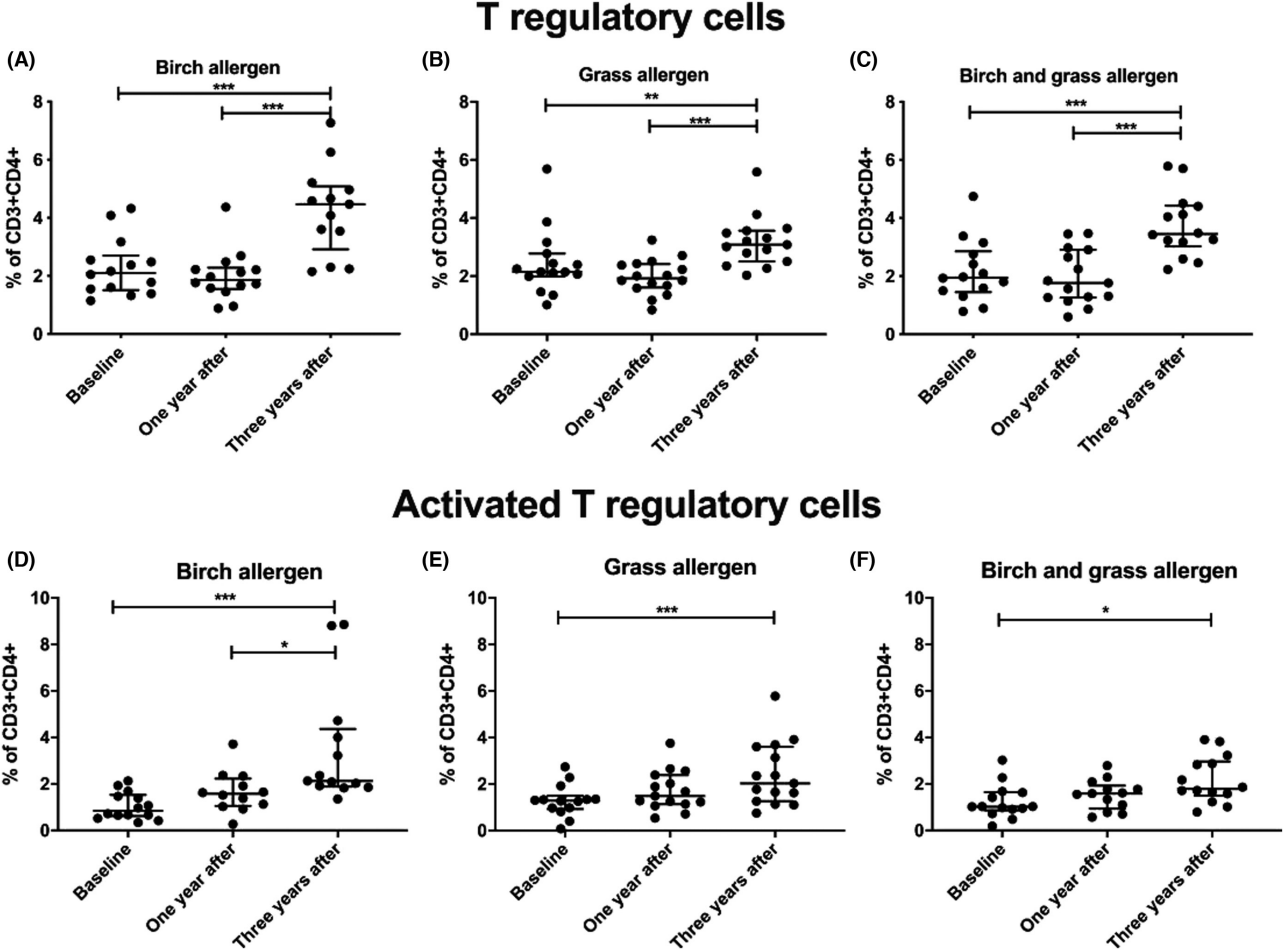
Proportion (%) of the T regulatory (Treg) cells in the CD3^+^CD4^+^ populations after intralymphatic immunotherapy with birch and/or 5‐grass allergen. Blood samples were collected at three time‐points: screening, 1 year after and 3 years after treatment had finished. The proportion of Treg cells, defined as CD4^dim^CD25^hi^FoxP3^+^ cells, increased over time in all treatment groups (A–C). The same trend was observed in the activated Treg cell population, defined as CD3^+^CD4^+^CD45RA^−^FoxP3^++^ (D–F). *p < .05; **p < .01; ***p <.001 from Wilcoxon signed rank tests. The lines indicate median and interquartile range (IQR, 25th and 75th percentile values). Only patients randomized in Linköping included

Flow cytometry data revealed that the proportion of Th1 cells, defined as CD3^+^CD4^+^CD45RA^−^Tbet^+^ cells, decreased between baseline and 3 years after treatment in the groups receiving ILIT with birch and placebo and 5‐grass and placebo (*p* < .05, Figure 3A,B). A significant increase was observed after 1 year in the group receiving birch and 5‐grass treatment and it seemed to decrease, but not significantly so, to 3 years (*p* < .05, Figure 3C). The proportion of Th2 cells, defined as CD3^+^CD4^+^CD45RA^−^GATA3^+^ cells, increased 3 years after treatment in the group receiving birch and placebo ILIT (Figure 3D, *p* < .01) and in the group receiving both treatments (Figure 3F, *p* < .05). This change was not significant in the group that received 5‐grass and placebo ILIT (Figure 3E). The proportion of Th17 cells, defined as CD3^+^CD4^+^ CD45RA^−^ RORC^+^ cells, decreased from baseline to 3 years post ILIT, independent of treatment (Figure 3G–I, *p* < .05). A significant reduction in Th17 cell frequencies was also observed from 1 year to 3 years after ILIT (*p* < .01). The Th17 memory population was significantly higher at baseline in the birch and placebo group than in the other treatment groups (*p* < .001, see File S5a). One year after treatment, a higher proportion of Th17 memory cells was observed in patients receiving 5‐grass and placebo ILIT than in the birch and placebo group (*p* < .05, see File S5b). No significant differences between the treatment groups were observed at 3 years (see File S5). The proportion of CD4^dim^CD25^hi^FoxP3^+^ Tregs (Figure 4A–C) and activated Tregs, defined as CD3^+^CD4^+^CD45RA^−^FoxP3^++^ cells (Figure 4D–F), significantly increased between baseline and 3 years, independent of treatment. In contrast, the proportion of resting Tregs, defined as CD3^+^CD4^+^CD45RA^+^FoxP3^+^ cells, was not affected by ILIT (data not shown).

### 3.6 Allergen‐induced cytokine and chemokine production

**FIGURE 5:**
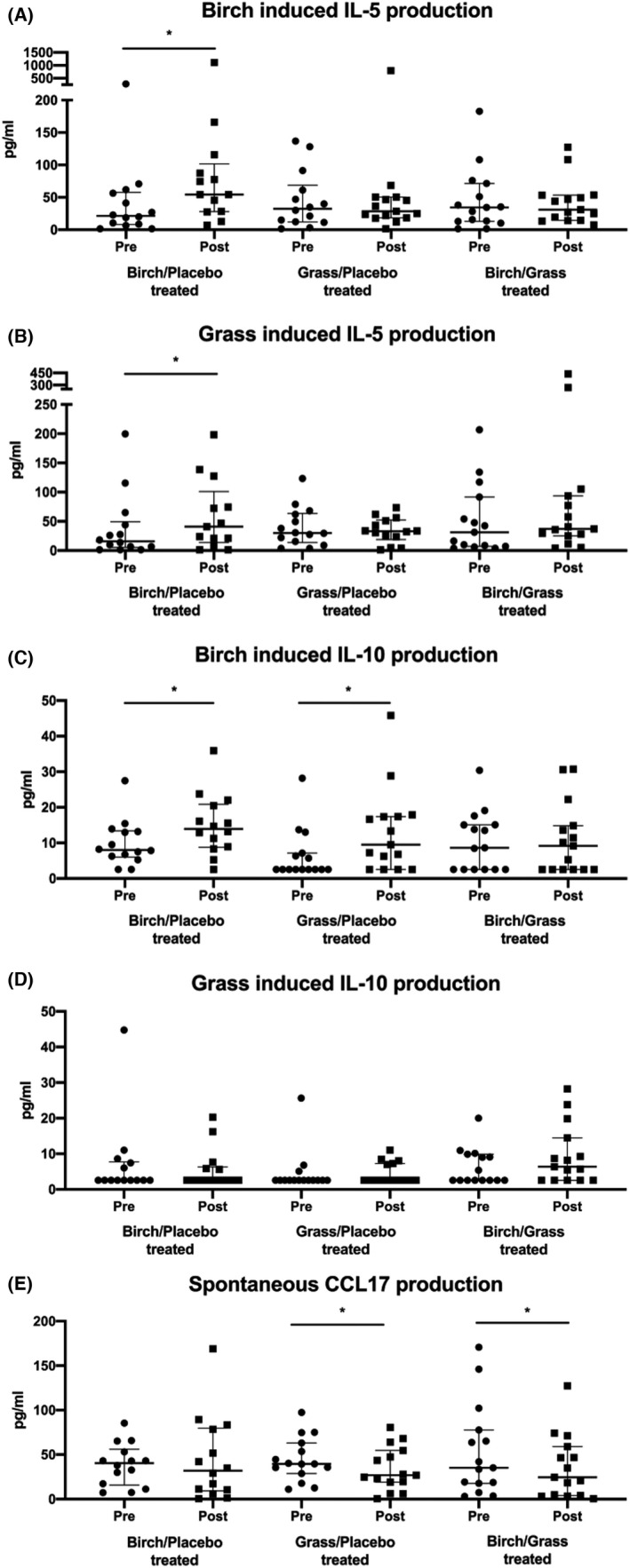
Spontaneous and allergen‐induced cytokine and chemokine production after intralymphatic immunotherapy with birch and/or 5‐grass. (A) birch‐induced IL‐5 production (B) grass induced IL‐5 production, (C) birch‐induced IL‐10 production, (D) grass induced IL‐10 production and (E) spontaneous CCL17 production. *p < .05 from Wilcoxon signed rank tests. The lines indicate median and interquartile range (IQR, 25th and 75th percentile values). Only patients randomized in Linköping included

At baseline and 1 year post ILIT, peripheral blood mononuclear cells were harvested and stimulated with birch and grass allergen *in vitro* to measure cytokine and chemokine secretion (see Files S6 and S7). Both birch and grass allergens induced an increase in IL‐5 production after birch and placebo ILIT (*p* < .05, Figure 5A,B), while IL‐5 secretion did not change in the other treatment groups. Increased birch allergen‐induced IL‐10 secretion was also observed after birch and placebo ILIT and 5‐grass and placebo ILIT, whereas no significant change occurred in the birch and 5‐grass group (Figure 5C). ILIT did not affect grass‐allergen IL‐10 production (Figure 5D). The spontaneous production of the CCL17 chemokine decreased after 5‐grass and placebo ILIT and birch and 5‐grass ILIT but not after birch and placebo ILIT (*p* < .05 Figure 5E).

## 4 DISCUSSION

The current study is to our knowledge the largest double‐blind randomized clinical ILIT trial to date. Patients with hayfever due to sensitization to both grass and birch pollen allergens received 5‐grass and birch ILIT, 5‐grass ILIT (with birch placebo), or birch ILIT (with grass placebo), thus with ‘active allergen placebo’ as suggested in the ARIA‐GA^2^LEN statement 2011.^29^ The study revealed statistically significant clinical efficacy of ILIT in all three treatment groups. Participants reported improvements in RTSS, RQLQ as well as reduced MS in the first pollen season following ILIT. The effects were sustained throughout the following three follow‐up seasons. We expected that the clinical efficacy of ILIT would mostly be observed for the targeted allergen, that is that patients receiving birch ILIT would respond better during the birch pollen season thanduring the grass pollen season and vice versa for patients with 5‐grass ILIT. However, patients receiving birch ILIT or 5‐grass ILIT reported similar improved clinical symptoms during pollen seasons for which they had received no treatment. This bystander effect has previously been hypothesized in SCIT both clinically and immunologically^13^, ^14^, ^15^ but not previously shown in ILIT. The clinical data were accompanied and supported by non‐allergen‐specific immunological changes such as an increase in the proportion of activated Treg cells in all treatment groups. Birch allergen‐induced secretion of IL‐10 was increased in both the birch/placebo and the grass/placebo group irrespectively of the allergen after the treatment. Furthermore, a decreased spontaneous production of the Th2 chemokine CCL17 was seen in the single treatment groups. In contrast, the increase in the number of Th2 cells and the increased level of allergen‐specific IL‐5 production after birch ILIT were unexpected. Thus, the beneficial clinical responses correlated with the increase in the frequency of Tregs and increased secretion of IL‐10. This potentially inhibitory bystander effect of Tregs and IL‐10 might explain why the improved clinical outcome was not dependent on the ILIT allergen and corroborates previously reported bystander immunological effects of SCIT in animal studies and one case report^13^, ^14^, ^15^

As clinical improvement was sustained throughout our study, we suggest the results are not only due to the placebo used in the single treatment groups, although placebo effects may be strong in AIT‐clinical trials.^30^ The positive clinical effect did not seem to be mediated by allergen‐specific IgG4, as these antibody levels were not clearly elevated between pre‐ILIT baseline and any of the time‐points thereafter. AIT efficacy has, however, often been reported to correlate with increased IgG4 in SCIT and SLIT.^6^ Moreover, in previous grass‐ or birch‐pollen ILIT studies, only moderate or no changes in IgG4 were determined,^18^, ^19^, ^20^, ^21^, ^22^ whereas a significant increase in allergen‐specific IgG4 was determined after ILIT with cat dander allergen.^31^ However, different results regarding IgG4 levels in SCIT, SLIT and ILIT may depend on the route of administration of the allergen.

The birch pollen counts in southeast Sweden in 2014 and 2018 were higher than in 2015, 2016, and 2017 (see File S8). As 55 study subjects received ILIT in 2014 and seventeen received ILIT in 2015, differences in pollen count were levelled out. Patients who received ILIT in 2015 compared well in the following seasons to patients that received ILIT in 2014. There were no differences in timing of blood samples. The patients recorded their RQLQ, RTSS and MS directly after the birch and grass pollen seasons, which are quite separate in Sweden, after enrolment, before ILIT and 3 years after, *that is* 4 years in all. This is a limitation, which may have caused a recall bias. However, the use of daily combined symptom medical score was not yet recommended when we designed this study. Of seventy‐two patients receiving ILIT, only two was lost to follow‐up after 3 years.

AEs were very common (>1/10) but most were judged as mild or moderate, generally at the allergen injection site (Table 3). These are also normal reactions after SCIT.^32^, ^33^ However, whereas patients receiving ILIT in the current study reported between none to three AEs, the number of AEs after SCIT can be up to 40–50. In a large study that analysed AEs in 1700 patients who had received SCIT, systemic AEs were reported in 3.3% of the patients and in 1.56/1000 injections.^33^ Oedema and pruritus at the injection site, flush, urticaria, wheezing, dyspnoea, eye pruritus, headache and abdominal pain are common (1–10%) or very common (>10%) with SCIT, whereas oral pruritus, oral oedema, rhinitis, headache, ear pruritus, throat irritation, asthma, abdominal pain, urticaria and fatigue are common or very common with SLIT.^34^ ILIT seems to give AEs similar to those with SCIT, but because ILIT only needs three injections, the AEs can be reduced by up to 90% compared with SCIT.

One limitation of this study is that it had no true placebo group because all the participants received ILIT against, at least, one pollen. However, the efficacy of ILIT have been described in earlier studies,^16^, ^17^, ^18^, ^19^, ^20^, ^21^, ^23^, ^35^, ^36^ but not whether there is a difference in response to treatment with one or two allergen in dual allergic patients.

AIT renders significant improvements in rhinoconjunctivitis and conjunctival sensitivity that persist at least 7 years after termination of treatment.^7^ In addition, AIT can prevent development of asthma^37^, ^38^ and new sensitizations in mono‐sensitized patients.^12^, ^39^ After an updosing phase of 7–15 weeks in SCIT, a maintenance phase of 3 years with injections every six to eight weeks is typically required to render long‐term tolerance. For SLIT, 55–82% of patients were reported to abandon the treatment before completing the recommended course of therapy.^40^ With only three injections over eight weeks, ILIT may overcome the disadvantages of long duration of treastment and poor compliance.

The results of this study add to the hitherto positive studies and suggest that ILIT may be effective and safe as treatment for pollen allergy. We will follow the patients in this study for symptoms, health‐related quality of life and adverse events in an open study until 2024 (EPN 2017/302‐31). ILIT may be an opportunity to make AIT more easily accessible to patients at a lower cost and less risk. However, there is a need for further studies to establish the optimal dose for efficacy and side effects.^25^

## 5 CONCLUSIONS

Intralymphatic immunotherapy against grass and birch pollen allergy was safe and seems effective. The unspecific effect of one allergen ILIT may be associated with bystander immune modulatory responses.

## CONFLICT OF INTEREST

LA has received honoraria as a speaker and/or adviser from AstraZeneca, Meda, Takeda, Teva, Boehringer Ingelheim, MSD, Sanofi, GlaxsSmithKleine, Novartis and Airsonett. JB has received honoraria as a speaker from ALK. PN has received honoraria as a speaker and/or adviser from AstraZeneca, CSL Behring and Takeda.

## AUTHOR CONTRIBUTIONS

The overall design of the study was conducted by LA, LN, UN, JB, MJ. The overall procurement of funds was provided by LA, LN, MJ.: LA, PR, UN were performed ILIT.: LA, JB, UN, PR, TT contributed to clinical evaluation and sample collection.: EA and MJ were designed the laboratory experiments.: EA, CA, GP, LP were performed the laboratory work. Statistical calculations were done by LA, EA, BR. The overall analysis of the data was done by LA, EA, JB, UN, MJ, LN, PN, PJ, KD. LA drafted the manuscript. All authors critically reviewed the manuscript.

## ETHICS STATEMENT

The study was approved by the Regional Ethics Committee in Linköping (EPN Dnr 2013/487‐31, amendments 2014/55‐32 and 2015/296‐31). Informed signed consent was obtained from the participants before inclusion.

## Supporting information

File S1

File S2

File S3

File S4

File S5

File S6

File S7

File S8

## Data Availability

The data sets used and/or analysed during the current study are available from the corresponding author on reasonable request.

## References

[1] Eriksson J , Ekerljung L , Ronmark E , et al. Update of prevalence of self‐reported allergic rhinitis and chronic nasal symptoms among adults in Sweden. Clin Respir J. 2012;6(3):159‐168. 21848956 10.1111/j.1752-699X.2011.00269.x

[2] Warm K , Hedman L , Lindberg A , Lotvall J , Lundback B , Ronmark E . Allergic sensitization is age‐dependently associated with rhinitis, but less so with asthma. J Allergy Clin Immunol. 2015;136(6):1559‐1565.e2. 26220530 10.1016/j.jaci.2015.06.015

[3] Newson RB , van Ree R , Forsberg B , et al. Geographical variation in the prevalence of sensitization to common aeroallergens in adults: the GA(2) LEN survey. Allergy. 2014;69(5):643‐651. 24654915 10.1111/all.12397

[4] Cardell LO , Olsson P , Andersson M , et al. TOTALL: high cost of allergic rhinitis – a national Swedish population‐based questionnaire study. NPJ Prim Care Respir Med. 2016;26:15082. 26845513 10.1038/npjpcrm.2015.82 PMC4741287

[5] Di Bona D , Bilancia M , Albanesi M , Caiaffa MF , Macchia L . Cost‐effectiveness of grass pollen allergen immunotherapy in adults. Allergy. 2020;75(9):2319‐2329. 32096242 10.1111/all.14246

[6] Akdis CA , Akdis M . Mechanisms of allergen‐specific immunotherapy and immune tolerance to allergens. World Allergy Organ J. 2015;8(1):17. 26023323 10.1186/s40413-015-0063-2 PMC4430874

[7] Roberts G , Pfaar O , Akdis CA , et al. EAACI Guidelines on Allergen Immunotherapy: allergic rhinoconjunctivitis. Allergy. 2018;73(4):765‐798. 28940458 10.1111/all.13317

[8] Klimek L , Pfaar O , Bousquet J , Senti G , Kundig T . Allergen immunotherapy in allergic rhinitis: current use and future trends. Exp Rev Clin Immunol. 2017;13(9):897‐906. 10.1080/1744666X.2017.1333423 28532268

[9] Durham SR , Walker SM , Varga EM , et al. Long‐term clinical efficacy of grass‐pollen immunotherapy. N Engl J Med. 1999;341(7):468‐475. 10441602 10.1056/NEJM199908123410702

[10] Dhami S , Nurmatov U , Arasi S , et al. Allergen immunotherapy for allergic rhinoconjunctivitis: a systematic review and meta‐analysis. Allergy. 2017;72(11):1597‐1631. 28493631 10.1111/all.13201

[11] Arvidsson MB , Löwhagen O , Rak S . Effect of 2‐year placebo‐controlled immunotherapy on airway symptoms and medication in patients with birch pollen allergy. J Allergy Clin Immunol. 2002;109(5):777‐783. 11994699 10.1067/mai.2002.123868

[12] Durham SR , Penagos M . Sublingual or subcutaneous immunotherapy for allergic rhinitis? J Allergy Clin Immunol. 2016;137(2):339‐349.e10. 26853126 10.1016/j.jaci.2015.12.1298

[13] Ciprandi G . Clinical bystander effect exerted by allergen immunotherapy: a hypothesis. Eur Ann Allergy Clin Immunol. 2015;47(2):62‐63. 25781197

[14] Moldaver DM , Bharhani MS , Rudulier CD , Wattie J , Inman MD , Larché M . Induction of bystander tolerance and immune deviation after Fel d 1 peptide immunotherapy. J Allergy Clin Immunol. 2019;143(3):1087‐1099.e4. 29906527 10.1016/j.jaci.2018.03.023

[15] Navarro S , Lazzari A , Kanda A , et al. Bystander immunotherapy as a strategy to control allergen‐driven airway inflammation. Mucosal Immunol. 2015;8(4):841‐851. 25425267 10.1038/mi.2014.115 PMC5410219

[16] Senti G , Prinz Vavricka BM , Erdmann I , et al. Intralymphatic allergen administration renders specific immunotherapy faster and safer: a randomized controlled trial. Proc Natl Acad Sci USA. 2008;105(46):17908‐17912. 19001265 10.1073/pnas.0803725105 PMC2582048

[17] Patterson AM , Bonny AE , Shiels WE 2nd , Erwin EA . Three‐injection intralymphatic immunotherapy in adolescents and young adults with grass pollen rhinoconjunctivitis. Ann Allergy Asthma Immunol. 2016;116(2):168‐170. 26706294 10.1016/j.anai.2015.11.010

[18] Schmid JM , Nezam H , Madsen HH , Schmitz A , Hoffmann HJ . Intralymphatic immunotherapy induces allergen specific plasmablasts and increases tolerance to skin prick testing in a pilot study. Clin Transl Allergy. 2016;6:19. 27231527 10.1186/s13601-016-0107-x PMC4880857

[19] Hylander T , Larsson O , Petersson‐Westin U , et al. Intralymphatic immunotherapy of pollen‐induced rhinoconjunctivitis: a double‐blind placebo‐controlled trial. Respir Res. 2016;17:10. 26817454 10.1186/s12931-016-0324-9 PMC4728811

[20] Hellkvist L , Hjalmarsson E , Kumlien Georen S , et al. Intralymphatic immunotherapy with 2 concomitant allergens, birch and grass: a randomized, double‐blind, placebo‐controlled trial. J Allergy Clin Immunol. 2018;142(4):1338‐1341.e9. 29908212 10.1016/j.jaci.2018.05.030

[21] Ahlbeck L , Ahlberg E , Nystrom U , Bjorkander J , Jenmalm MC . Intralymphatic allergen immunotherapy against pollen allergy: a 3‐year open follow‐up study of 10 patients. Ann Allergy Asthma Immunol. 2018;121(5):626‐627. 30021119 10.1016/j.anai.2018.07.010

[22] Witten M , Malling HJ , Blom L , Poulsen BC , Poulsen LK . Is intralymphatic immunotherapy ready for clinical use in patients with grass pollen allergy? J Allergy Clin Immunol. 2013;132(5):1248‐1252.e5. 24035151 10.1016/j.jaci.2013.07.033

[23] Senti G , Crameri R , Kuster D , et al. Intralymphatic immunotherapy for cat allergy induces tolerance after only 3 injections. J Allergy Clin Immunol. 2012;129(5):1290‐1296. 22464647 10.1016/j.jaci.2012.02.026

[24] Lee SP , Choi SJ , Joe E , et al. A pilot study of intralymphatic immunotherapy for house dust mite, cat, and dog allergies. Allergy Asthma Immunol Res. 2017;9(3):272‐277. 28293934 10.4168/aair.2017.9.3.272 PMC5352579

[25] Senti G , Freiburghaus AU , Larenas‐Linnemann D , et al. Intralymphatic immunotherapy: update and unmet needs. Int Arch Allergy Immunol. 2019;178(2):141‐149. 30391954 10.1159/000493647

[26] Devillier P , Chassany O , Vicaut E , et al. The minimally important difference in the Rhinoconjunctivitis Total Symptom Score in grass‐pollen‐induced allergic rhinoconjunctivitis. Allergy. 2014;69(12):1689‐1695. 25155425 10.1111/all.12518

[27] Juniper EF , Thompson AK , Ferrie PJ , Roberts JN . Validation of the standardized version of the Rhinoconjunctivitis Quality of Life Questionnaire. J Allergy Clin Immunol. 1999;104(2 Pt 1):364‐369. 10452758 10.1016/s0091-6749(99)70380-5

[28] Fauquert J‐L , Jedrzejczak‐Czechowicz M , Rondon C , et al. Conjunctival allergen provocation test : guidelines for daily practice. Allergy. 2017;72(1):43‐54. 27430124 10.1111/all.12986

[29] Bousquet J , Schünemann HJ , Bousquet PJ , et al. How to design and evaluate randomized controlled trials in immunotherapy for allergic rhinitis: an ARIA‐GA(2) LEN statement. Allergy. 2011;66(6):765‐774. 21496059 10.1111/j.1398-9995.2011.02590.x

[30] Pfaar O , Agache I , Bergmann K , et al. Placebo effects in allergen immunotherapy – an EAACI Task Force Position Paper. Allergy. 2021;76:629‐647. 32324902 10.1111/all.14331

[31] Freiberger SN , Zehnder M , Gafvelin G , Grönlund H , Kündig TM , Johansen P . IgG4 but no IgG1 antibody production after intralymphatic immunotherapy with recombinant MAT‐Feld1 in human. Allergy. 2016;71(9):1366‐1370. 27253988 10.1111/all.12946

[32] Kowalski ML , Ansotegui I , Aberer W , et al. Risk and safety requirements for diagnostic and therapeutic procedures in allergology: World Allergy Organization Statement. World Allergy Organ J. 2016;9(1):33. 27777642 10.1186/s40413-016-0122-3 PMC5062928

[33] Schiappoli M , Ridolo E , Senna G , et al. A prospective Italian survey on the safety of subcutaneous immunotherapy for respiratory allergy. Clin Exp Allergy. 2009;39(10):1569‐1574. 19486027 10.1111/j.1365-2222.2009.03286.x

[34] Aasbjerg K , Dalhoff KP , Backer V . Adverse events during immunotherapy against grass pollen‐induced allergic rhinitis – differences between subcutaneous and sublingual treatment. Basic Clin Pharmacol Toxicol. 2015;117(2):73‐84. 25968654 10.1111/bcpt.12416

[35] Hylander T , Latif L , Petersson‐Westin U , Cardell LO . Intralymphatic allergen‐specific immunotherapy: an effective and safe alternative treatment route for pollen‐induced allergic rhinitis. J Allergy Clin Immunol. 2013;131(2):412‐420. 23374268 10.1016/j.jaci.2012.10.056

[36] Konradsen JR , Grundström J , Hellkvist L , et al. Intralymphatic immunotherapy in pollen‐allergic young adults with rhinoconjunctivitis and mild asthma: a randomized trial. J Allergy Clin Immunol. 2020;145(3):1005‐1007.e7. 31775016 10.1016/j.jaci.2019.11.017

[37] Jacobsen L , Niggemann B , Dreborg S , et al. Specific immunotherapy has long‐term preventive effect of seasonal and perennial asthma: 10‐year follow‐up on the PAT study. Allergy. 2007;62(8):943‐948. 17620073 10.1111/j.1398-9995.2007.01451.x

[38] Valovirta E , Petersen TH , Piotrowska T , et al. Results from the 5‐year SQ grass sublingual immunotherapy tablet asthma prevention (GAP) trial in children with grass pollen allergy. J Allergy Clin Immunol. 2018;141(2):529‐538.e13. 28689794 10.1016/j.jaci.2017.06.014

[39] Pajno GB , Barberio G , De Luca F , Morabito L , Parmiani S . Prevention of new sensitizations in asthmatic children monosensitized to house dust mite by specific immunotherapy. A six‐year follow‐up study. Clin Exp Allergy. 2001;31(9):1392‐1397. 11591189 10.1046/j.1365-2222.2001.01161.x

[40] Bender BG , Oppenheimer J . The special challenge of nonadherence with sublingual immunotherapy. J Allergy Clin Immunol Pract. 2014;2(2):152‐155. 24607041 10.1016/j.jaip.2014.01.003

