# Supplementary material for: Intralymphatic immunotherapy with one or two allergens renders similar clinical response in patients with allergic rhinitis due to birch and grass pollen
Source: Clin Exp Allergy. 2022 Apr 1;52(6):747–59. doi: 10.1111/cea.14138 (PMC9325375; doi:10.1111/cea.14138)
Supplement: Supplementary file 1 — File S1 [file CEA-52-747-s007.docx]

**Additional file 1**

**Medication Score from the Swedish Association for Allergology 2011**

|  | **Never** | **Occasionally** | **Daily** |
| --- | --- | --- | --- |
| Oral antihistamine | 0p | 1p | 2p |
| Local treatment nose, except steroids | 0p | 1p | 2p |
| Inhaled bronchodilator | 0p | 1p | 1p |
| Nasal steroids | 0p | 2p | 4p |
| Other (*i.e.* montelukast, theophylline) | 0p | 2p | 4p |
| Peroral or ocular steroids | 0p | 4p | 8p |
| Steroid injection | 0p | 4p | - |
| Omalizumab | 0p | 8p | - |
