# Supplementary material for: Intralymphatic immunotherapy with one or two allergens renders similar clinical response in patients with allergic rhinitis due to birch and grass pollen
Source: Clin Exp Allergy. 2022 Apr 1;52(6):747–59. doi: 10.1111/cea.14138 (PMC9325375; doi:10.1111/cea.14138)
Supplement: Supplementary file 2 — File S2 [file CEA-52-747-s002.docx]

**Additional file 2**

**Immune laboratory methods**

**Flow cytometry**

Flow cytometry was used to analyze peripheral T helper cell populations in whole blood drawn in EDTA tubes at randomization, one and three years after treatment had finished. Within the lymphocyte population (based on FSC and SSC), the Th population was defined using PECy7-conjugated anti-CD4 (SK3) and APCCy7-conjugated anti-CD3 (SK7) antibodies (BD Biosciences, San Jose, CA, USA). Within the CD3+CD4+ population, the naïve and memory cell populations were determined using v450-conjugated anti-CD45RA (HI100) antibodies (BD Biosciences). Expression of cellular Th cell lineage markers was determined using PE-conjugated anti-GATA3 (TWAJ), eFlour 660-conjugated anti-Tbet (eBio4B10), PE-conjugated anti-RORC (AFKJS-9), PerCP-Cy5.5-conjugated anti-CD25 (M-A251) and FITC-conjugated anti-Foxp3 (PCH101) antibodies (eBiosciences, San Diego, CA, USA).The proportion of Th subsets was determined using the baseline of 1% positive cells in the CD45RA^+^ naïve (undifferentiated) and the same gate settings were then transferred to the CD45RA^-^ memory (differentiated) CD3^+^CD4^+^ Th cell population. Treg cells were defined as CD4^dim^CD25^hi^Foxp3^+^ and subtypes of Treg cells were subdivided into CD3^+^CD4^+^CD45RA^+/-^Foxp3^+/++^, *i.e.* resting and activated Tregs (37), respectively. Complete gating strategy can be found in Additional file 3. Data were acquired on a BD FACS CANTO II and analyzed using Kaluza 1.2 software (Beckman Coulter).

**Cell cultures stimulated with birch and grass allergen**

Venous blood was drawn from the patients at randomization, and one year after treatment had finished into heparin-treated tubes (Vacuette, Greiner Labortechnik, Kremsmünster, Austria). Peripheral blood mononuclear cells were isolated on a Ficoll Plaque density gradient (Sigma-Aldrich, Stockholm, Sweden) as described in detail elsewhere (38). Aliquots of 1 ml, containing 1 x 10^6^ cells, were stimulated with birch or timothy Aquagen allergen extracts (ALK, Hørsholm, Denmark) at 10,000 SQ-U/mL for 6 days at 37°C with 5% CO_2_ in AIM-V serum-free medium (Life Technologies AB, Täby, Sweden) with 20 μM β-mercaptoethanol (Sigma-Aldrich). The supernatants were collected after centrifugation at 400 x g for 5 min and stored at –70°C until further analysis.

**Quantification of IL-5, IL-10, IL-13, IFN-γ, CCL17 and CXCL10 with Luminex**

Immunological changes, including T helper cell associated cytokines and chemokine production after allergen stimulation, were analyzed using an in-house multiplex Luminex assay, as previously described (39) .The lower detection limit for IL-5 was 2.82 pg/mL, for IL-10 5.1 pg/mL, for IL-13 76.08 pg/mL, for IFN-γ 25.36 pg/mL, for CCL17 1.17 pg/mL and for CXCL10 30.4 pg/mL. Background responses with medium only were subtracted from the allergen-induced responses. If the responses were under the limit, they were given half the value of the detection limit. The majority of the CXCL10 levels were above the upper detection limit and were re-analyzed diluted 1:10 or 1:100.
