# Supplementary material for: Intralymphatic immunotherapy with one or two allergens renders similar clinical response in patients with allergic rhinitis due to birch and grass pollen
Source: Clin Exp Allergy. 2022 Apr 1;52(6):747–59. doi: 10.1111/cea.14138 (PMC9325375; doi:10.1111/cea.14138)
Supplement: Supplementary file 3 — File S3 [file CEA-52-747-s001.docx]

**Additional file 3**

**Gating strategy**


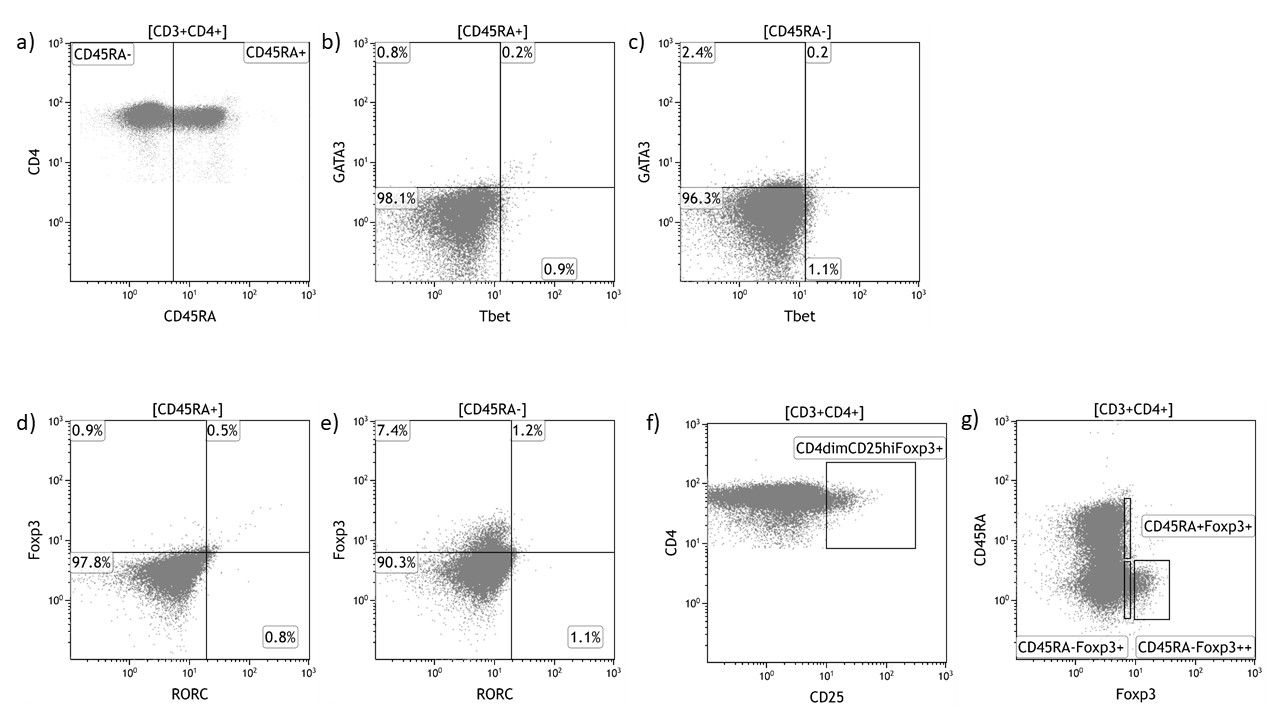
Gating strategy to identify T helper cell populations from flow cytometry analysis. A gate was set to isolate the lymphocyte population by measuring forward scatter (FSC) on the X axis (size) and side scatter (SSC) on the Y axis (granularity). To define T lymphocytes, a gate was set for CD3^+^CD4^+^ cells. The lymphocyte gate was also used to define CD3^+^CD4^+^ T helper (Th) cells. a) Naïve (CD45RA^+^) and memory (CD45RA^-^) Th cells were defined by their expression of CD45RA. b) To define Th1 and Th2 cells, the populations were gated on expression of the intracellularly expressed Th cell lineage markers, T-box expressed in T cells (Tbet), a transcription factor expressed by Th1, and GATA binding protein 3 (GATA3), a key transcription factor in Th2 cells. Cells in the naïve population were not expected to express the markers, and a gate was set in that population (maximum 1% and minimum 0.6% cells positive for the markers). In the memory population (c) The gate from the naïve population was used to define cells expressing Tbet and GATA3. d) and e) A similar strategy was used to define Th17 cells as RORC^+^ CD45RA^-^ cells. f) T regulatory cells were defined as CD4^dim^CD25^hi^Foxp3^+^ cells. g) In addition, two T regulatory subpopulations were isolated from the CD3^+^CD4^+^ Th cell population, depending on their expression of the transcription factor forkhead box P3 (FoxP3) and CD45RA. CD3^+^CD4^+^CD45RA^+/-^Foxp3^+/++^, *i.e.* resting and activated Tregs, respectively
