# Supplementary material for: Intralymphatic immunotherapy with one or two allergens renders similar clinical response in patients with allergic rhinitis due to birch and grass pollen
Source: Clin Exp Allergy. 2022 Apr 1;52(6):747–59. doi: 10.1111/cea.14138 (PMC9325375; doi:10.1111/cea.14138)
Supplement: Supplementary file 4 — File S4 [file CEA-52-747-s008.docx]

**Additional file 4**

**Results**: Quality of life (RQLQ), symptoms (RTSS), medication (MS), levels of allergen specific IgE and IgG4, skin prick test (SPT) and conjunctival allergen provocation test (CAPT)

| **RQLQ birch season**  **Median [IQR]** | **Treated with birch and 5-grass** | **Treated with birch and placebo** | **Treated with 5-grass and placebo** |
| --- | --- | --- | --- |
| Before treatment | 2.84 [2.46-3.90] | 3.14 [2.75–4.08] | 3.30 [2.50-3.85] |
| 1^st^ year after treatment | 1.89 [0.54-2.46] | 1.54 [0.60-2.47] | 1.39 [0.55-2,58] |
| 2^nd^ year after treatment | 1.21 [0.46-2.48] | 1.36 [0.68-2.11] | 1.43 [1.14-2,07] |
| 3^rd^ year after treatment | 1.23 [0.46-2.53] | 1.23 [0.47-2.03] | 1.70 [1.19-2,42] |
| p-value (Before - 3^rd^) | 0.01 | 0.002 | 0.01 |

| **RQLQ grass season**  **Median [IQR]** | **Treated with birch and 5-grass** | **Treated with birch and placebo** | **Treated with 5-grass and placebo** |
| --- | --- | --- | --- |
| Before treatment | 3.12 [2.05-3.91] | 2.68 [1.82-3.50] | 2.91 [2.45-3.54] |
| 1^st^ year after treatment | 2.11 0.61-2.93] | 1.21 [0.33-2.48] | 1.52 [0.67-2.48] |
| 2^nd^ year after treatment | 1.59 [0.67-2.66] | 0.93 [0.43-1.39] | 1.64 [0.82-2.07] |
| 3^rd^ year after treatment | 1.18 [0.43] | 0.80 [0.38-1.62] | 1.30 [0.79-2.13] |
| p value (Before - 3^rd^) | <0.001 | 0.004 | <0.001 |

| **RTSS birch season**  **Median [IQR]** | **Treated with birch and 5-grass** | **Treated with birch and placebo** | **Treated with 5-grass and placebo** |
| --- | --- | --- | --- |
| Before treatment | 11.00 [9.00-14.00] | 14.00 [8.50-15.00] | 13.00 [10.25-15.00] |
| 1^st^ year after treatment | 6.00 [3.00-9.00] | 8.00 [5.00-12.00] | 6.00 [3.25-8.00] |
| 2^nd^ year after treatment | 7.00 [3.00-11.00] | 7.00 [5.00-12.00] | 7.00 [5.75-9.25] |
| 3^rd^ year after treatment | 5.50 [3.00-10.00] | 7.50 [4.00-11.00] | 7.50 [4.50-11.00] |
| p value (Before - 3^rd^) | <0.001 | 0.01 | 0.01 |

| **RTSS grass season**  **Median [IQR]** | **Treated with birch and 5-grass** | **Treated with birch and placebo** | **Treated with 5-grass and placebo** |
| --- | --- | --- | --- |
| Before treatment | 11.00 [9.00-14.00] | 11.50 [6.50-15.00] | 11.50 [9.00-14.00] |
| 1^st^ year after treatment | 8.00 [4.00-12.50] | 6.00 [3.00-10.00] | 8.00 [3.25-9.75] |
| 2^nd^ year after treatment | 7.00 [2.00-12.00] | 5.00 [3.00-8.00] | 7.00 [4.00-9.50] |
| 3^rd^ year after treatment | 6.00 [4.00-10.25] | 6.00 [2.25-10.00] | 7.00 [3.25-9.00] |
| p value (Before - 3^rd^) | 0.01 | 0.006 | <0.001 |

| **MS birch season**  **Median [IQR]** | **Treated with birch and 5-grass** | **Treated with birch and placebo** | **Treated with 5-grass and placebo** |
| --- | --- | --- | --- |
| Before treatment | 9.00 [6.00-12.75] | 10.00 [5.25-11.75] | 8.00 [7.00-11.25] |
| 1^st^ year after treatment | 3.00 [0.25-5.75] | 4.00 [1.00-7.00] | 2.50 [0.00-8.50] |
| 2^nd^ year after treatment | 4.00 [0.00-6.00] | 3.00 [1.00-8.00] | 3.00 [1.00-8.25] |
| 3^rd^ year after treatment | 4.00 [1.50-6.00] | 3.00 [2.00-7.75] | 4.00 [2.25-7.25] |
| p value (Before - 3^rd^) | 0.01 | <0.001 | 0.03 |

| **MS grass season**  **Median [IQR])** | **Treated with birch and 5-grass** | **Treated with birch and placebo** | **Treated with 5-grass and placebo** |
| --- | --- | --- | --- |
| Before treatment | 8.50 [6.00-12.00] | 8.50 [5.00-11.00] | 8.00 [6.00-11.50] |
| 1^st^ year after treatment | 4.00 [1.00-6.00] | 3.00 [2.00-5.00] | 2.50 [0.00-6.75] |
| 2^nd^ year after treatment | 4.00 [0.00-8.00] | 2.00 [0.00-5.00] | 3.00 [0.75-6.50] |
| 3^rd^ year after treatment | 4.00 [2.00-6.00] | 4.00 [1.25-5.75] | 3.50 [1.25-5.00] |
| p value (Before - 3^rd^) | 0.01 | 0.002 | 0.01 |

| **IgE birch (kU/L)**  **Mean (SD)** | **Treated with birch and 5-grass** | **Treated with birch and placebo** | **Treated with 5-grass and placebo** |
| --- | --- | --- | --- |
| Before treatment | 21.17 (20.54) | 25.45 (27.13) | 33.18 (34.25) |
| 1^st^ year after treatment | 15.33 (20.71) | 21.50 (25.87) | 22.21 (23.05) |
| 2^nd^ year after treatment | - | - | - |
| 3^rd^ year after treatment | 16.20 (22.02) | 17.71 (22.58) | 24.42 (24.79) |
| p value (Before - 3^rd^) | 0.06 | 0.004 | 0.004 |

| **IgE timothy (kU/L)**  **Mean (SD)** | **Treated with birch and 5-grass** | **Treated with birch and placebo** | **Treated with 5-grass and placebo** |
| --- | --- | --- | --- |
| Before treatment | 17.81 (23.61) | 16.60 (22.58) | 17.39 (23.66) |
| 1^st^ year after treatment | 21.53 (22.56) | 14.73 (22.33) | 18.51 (23.98) |
| 2^nd^ year after treatment | - | - | - |
| 3^rd^ year after treatment | 13.56 (19.07) | 11.00 (12.77) | 14.60 (21.41) |
| p value (Before - 3^rd^) | 1.00 | 0.02 | 0.11 |

| **IgE total (kU/L)**  **Mean (SD)** | **Treated with birch and 5-grass** | **Treated with birch and placebo** | **Treated with 5-grass and placebo** |
| --- | --- | --- | --- |
| Before treatment | 150.14 (145,66) | 202.22 (420.20) | 200.50 (214.02) |
| 1^st^ year after treatment | 146.05 (148.00) | 115.04 (120.04) | 196.18 (243.56) |
| 2^nd^ year after treatment | - | - | - |
| 3^rd^ year after treatment | 150.00 (173.61) | 97.87 (67.15) | 165.32 (163.69) |
| p value (Before - 3^rd^) | 0.92 | 0.20 | 0.45 |

| **IgG4 birch (mg/L)**  **Mean (SD)** | **Treated with birch and 5-grass** | **Treated with birch and placebo** | **Treated with 5-grass and placebo** |
| --- | --- | --- | --- |
| Before treatment | 0.51 (0.37) | 0.58 (1.05) | 1.97 (5.79) |
| 1^st^ year after treatment | 0.52 (0.39) | 0.44 (0.53) | 1.32 (3.25) |
| 2^nd^ year after treatment | - | - | - |
| 3^rd^ year after treatment | 0.54 (0.38) | 0.38 (0.61) | 1.61 (4.80) |
| p value (Before - 3^rd^) | 0.92 | 0.07 | 0.08 |

| **IgG4 timothy (mg/L)**  **Mean (SD)** | **Treated with birch and 5-grass** | **Treated with birch and placebo** | **Treated with 5-grass and placebo** |
| --- | --- | --- | --- |
| Before treatment | 0.36 (0.23) | 0.33 (0.61) | 0.75 (1.96) |
| 1^st^ year after treatment | 0.50 (0.36) | 0.34 (0.38) | 0.79 (1.91) |
| 2^nd^ year after treatment | - | - | - |
| 3^rd^ year after treatment | 0.44 (0.30) | 0.22 (0.21) | 0.66 (1.53) |
| p value (Before - 3^rd^) | 0.02 | 0.07 | 0.20 |

| **SPT birch (mm)**  **Mean (SD)** | **Treated with birch and 5-grass** | **Treated with birch and placebo** | **Treated with 5-grass and placebo** |
| --- | --- | --- | --- |
| Before treatment | 5.90 (2.26) | 5.96 (2.29) | 6.15 (2.14) |
| 1^st^ year after treatment | 5.54 (2.99) | 6.09 (1.92) | 5.33 (1.89) |
| 2^nd^ year after treatment | 5.59 (2.38) | 6.10 (3.32) | 5.81 (2.04) |
| 3^rd^ year after treatment | 6.04 (2.62) | 6.65 (2.62) | 5.19 (2.01) |
| p value (Before - 3^rd^) | 0.79 | 0.38 | 0.19 |

| **SPT timothy (mm)**  **Mean (SD)** | **Treated with birch and 5-grass** | **Treated with birch and placebo** | **Treated with 5-grass and placebo** |
| --- | --- | --- | --- |
| Before treatment | 7.63 (3.26) | 5.46 (3.14) | 6.19 (2.08) |
| 1^st^ year after treatment | 8.31 (4.27) | 6.23 (2.93) | 6.78 (2.44) |
| 2^nd^ year after treatment | 7.78 (3.46) | 6.75 (3.03) | 6.58 (2.51) |
| 3^rd^ year after treatment | 6.43 (2.66) | 6.23 (2.53) | 6.19 (2.41) |
| p value (Before - 3^rd^) | 0.12 | 0.38 | 0.19 |

| **CAPT timothy**  **Mean (SD)** | **Treated with birch and 5-grass** | **Treated with birch and placebo** | **Treated with 5-grass and placebo** |
| --- | --- | --- | --- |
| Before treatment | 3.57 (1.16) | 4 (0.90) | 4.08 (1.06) |
| 1^st^ year after treatment | 4.13 (1.01) | 4.3 (1.22) | 4.46 (1.18) |
| 2^nd^ year after treatment | - | - | - |
| 3^rd^ year after treatment | nd | nd | nd |
| p value (Before – 1^st^) | 0.02 | 0.19 | 0.03 |
