# Supplementary material for: Intralymphatic immunotherapy with one or two allergens renders similar clinical response in patients with allergic rhinitis due to birch and grass pollen
Source: Clin Exp Allergy. 2022 Apr 1;52(6):747–59. doi: 10.1111/cea.14138 (PMC9325375; doi:10.1111/cea.14138)
Supplement: Supplementary file 5 — File S5 [file CEA-52-747-s005.docx]

**Additional file 5**

**Birch-induced cytokine and chemokine response** (pg/mL) after intralymphatic **immunotherapy. p* < 0.05 from Wilcoxon signed rank tests. All values are presented as** median and interquartile range (25^th^ and 75^th^ quartile values)

|  | **Birch treated** | | | **Grass treated** | | | **Birch and Grass treated** | | |
| --- | --- | --- | --- | --- | --- | --- | --- | --- | --- |
|  | **Pre** | **Post** | **P value*** | **Pre** | **Post** | **P value*** | **Pre** | **Post** | **P value*** |
| IL-5 | 21.41,  8.11 -  57.74 | 54.26,  27.98-  101.5 | **0.01** | 32.43,  11.95-  68.67 | 28.46,  18.44-  50.39 | 0.95 | 34.47,  12.83-  71.32 | 31.04,  14.46-  53.43 | 0.85 |
| IL-10 | 8.035,  6.018-  13.38 | 13.94,  8.78-  20.87 | **0.04** | 2.55,  2.55-  7.16 | 9.51,  2.55-  17.38 | **0.03** | 8.64,  2.55-  15.09 | 9.17,  2.55-  14.87 | 0.97 |
| IL-13 | 1281,  352.3-  2028 | 1582,  747.2-  2769 | 0.22 | 918,  562.6-  2329 | 983.8,  678.5-  1257 | 0.42 | 1011,  527.2-  2101 | 922.9,  370.9-  2193 | 0.85 |
| IFN$\gamma$ | 466.6,  155.8-  819.5 | 525.9,  175.8-  773.3 | 0.71 | 391.1,  261.4-  561 | 332.3,  172.3-  503 | 0.30 | 359.9,  262.3-  553.7 | 192.1,  127.3-  446.5 | 0.15 |
| CCL17 | 113.6,  15.36-  213.6 | 148.4,  7.8-  268.2 | 0.46 | 74.79,  28.98-  217 | 70.47,  24.04-  297.6 | 0.98 | 103.4,  13.46-  367.8 | 80.02,  30.61-  423.3 | 0.85 |
