# Supplementary material for: Intralymphatic immunotherapy with one or two allergens renders similar clinical response in patients with allergic rhinitis due to birch and grass pollen
Source: Clin Exp Allergy. 2022 Apr 1;52(6):747–59. doi: 10.1111/cea.14138 (PMC9325375; doi:10.1111/cea.14138)
Supplement: Supplementary file 6 — File S6 [file CEA-52-747-s006.docx]

**Additional file 6**

**Grass-induced cytokine and chemokine response** (pg/mL) after intralymphatic immunotherapy. p* < 0.05 from Wilcoxon signed rank tests. All values are presented as median and interquartile range (25^th^ and 75^th^ quartile values)

|  | **Birch treated** | | | **Grass treated** | | | **Birch and Grass treated** | | |
| --- | --- | --- | --- | --- | --- | --- | --- | --- | --- |
|  | **Pre** | **Post** | **P value*** | **Pre** | **Post** | **P value*** | **Pre** | **Post** | **P value*** |
| IL-5 | 15,87,  5.34-  49.25 | 40.88,  13.91-  101 | **0.04** | 30.07,  13.85-  63.58 | 33.06,  19-  52.35 | 0.8 | 31.41,  7.21-  91.63 | 37.03,  25.22-  93.6 | 0.19 |
| IL-10 | 2.55,  2.55-  7.74 | 2.55,  2.55-  6.3 | 0.84 | 2.55,  2.55-  2.55 | 2.55,  2.55-  7.28 | 0.63 | 2.55,  2.55-  9.9 | 6.4,  2.55-  14.48 | 0.07 |
| IL-13 | 476.5,  239.3-  1294 | 1135,  215.7-  2615 | 0.22 | 793.4,  342.9-  1480 | 593.9,  253-  1396 | 0.76 | 663.7,  377.2-  3215 | 1204,  519.5-  3646 | 0.22 |
| IFN$\gamma$ | 90.66,  12.68-  139.3 | 50.68,  23.82-  146.6 | 0.91 | 88.12,  25.77-  156.9 | 62.39,  36.79-  147.4 | >0.99 | 92.61,  12.68-  243.3 | 57.15,  12.68-  253.4 | 0.7 |
| CCL17 | 121.6,  75.36-  310.3 | 183.1,  52.04-  424.3 | 0.43 | 171,  60.08-  344.5 | 174.8,  30.7-  283.6 | 0.34 | 122.5,  51.23-  409.8 | 223.4,  64.57-  658.9 | 0.72 |
| CXCL10 | 921.3,  15.2-  4571 | 332,  15.2-  2347 | 0.85 | 197.6,  15.2-  2291 | 338.2,  15.2-  1966 | 0.41 | 2096,  415-  8572 | 537.7,  15.20-  3903 | 0.41 |
