# Supplementary material for: Intralymphatic immunotherapy with one or two allergens renders similar clinical response in patients with allergic rhinitis due to birch and grass pollen
Source: Clin Exp Allergy. 2022 Apr 1;52(6):747–59. doi: 10.1111/cea.14138 (PMC9325375; doi:10.1111/cea.14138)
Supplement: Supplementary file 7 — File S7 [file CEA-52-747-s004.docx]

**Additional file 7**


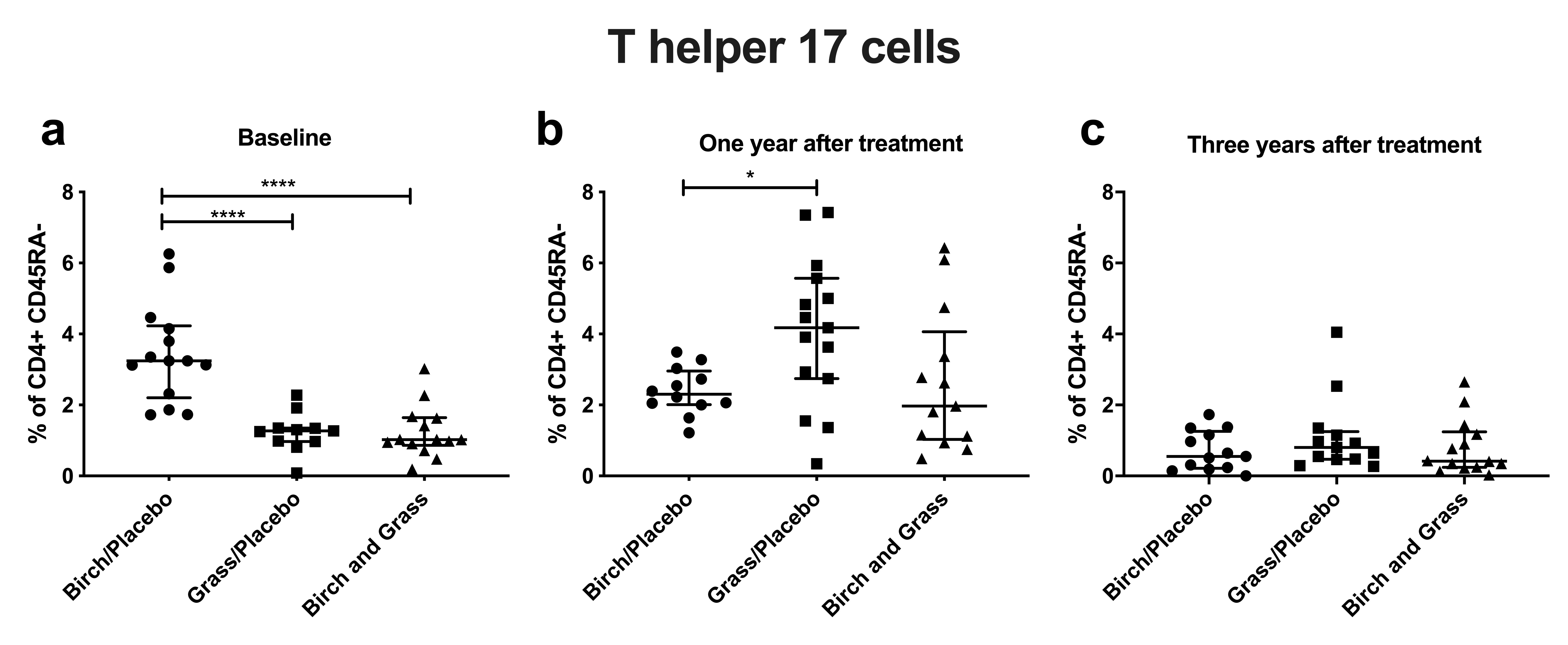


**Proportion (%) of the T helper (Th) 17** cell in the CD4+CD45RA memory population at each timepoint. a) Comparison of treatment groups at baseline, b) one year after treatment and c) three years after treatment had finished.

p* < 0.05; p**** < 0.0001 from unpaired Mann-Whitney U test. The lines indicate median and interquartile range (IQR, 25^th^ and 75^th^ percentile values). Only patients randomized in Linköping included.
